# Function Mechanism of Intellectual Property Capability on Relay Innovation Based on CWLBGSO-DAG-Bootstrap SEM: Mediating Effect of Knowledge Matching and Moderating Effect of Relationship Learning

**DOI:** 10.1155/2022/4357917

**Published:** 2022-06-28

**Authors:** Qiang Liu, Taimin Ding, Yafeng Li, Ziwei Gao, Yu Guo

**Affiliations:** ^1^School of Economics and Management, Liaoning University of Technology, Jinzhou 121001, China; ^2^School of Economics and Management, Harbin Engineering University, Harbin 150001, China

## Abstract

Taking unicorn enterprises as the research objects, this study constructs the conceptual model of function mechanism of intellectual property capability on relay innovation performance and introduces knowledge matching as the mediating variable and relationship learning as the moderating variable. Hybrid methods composed of fusion of rough set and binary firefly algorithm based on community weak connection mechanism (CWLBGSO), directed acyclic graph (DAG), and Bootstrap structural equation modeling (Bootstrap SEM) are used to carry out empirical analysis of the conceptual model of function mechanism. The empirical analysis results show that strong intellectual property capability has a positive role in promoting the relay innovation performance of unicorn enterprises, knowledge matching plays the positive mediating role between intellectual property capability and relay innovation, relationship learning plays the moderating role of the relationships between intellectual property capability and relay innovation, relationship learning plays the moderating role of the relationships between intellectual property capability and knowledge matching, and relationship learning plays the moderating role of the relationships between knowledge matching and relay innovation.

## 1. Introduction

In the new era of innovation-driven development, innovation has changed from the simple form based on industry to the common innovation form based on information and knowledge society. According to the 2017 Chinese unicorn enterprise development report and Zhongguancun unicorn enterprise development report, by the end of 2017, there had been 164 Chinese unicorn enterprises with a total valuation of US $624.9 billion, and well-known enterprises such as Ant Finance Services, Didi Travel, and Xiaomi Technology had emerged. These enterprises have the characteristics of explosive growth, such as strong innovation ability, short growth cycle, and large growth span, and have attracted more and more attention from all sectors of society. As the important member of emerging innovation subjects, unicorn enterprises promote the transformation of relevant industrial structure to the connotative development mode relying on knowledge accumulation, technological progress, and improving the quality of labor force. With the gradual deepening of information, knowledge, and technology interaction among innovation subjects, traditional innovation methods can no longer meet the overall innovation process of unicorn enterprises. New innovation modes such as deviant innovation, relay innovation, and subversive innovation are gradually respected by unicorn enterprises.

Relay innovation is typically characterized by “multiagent participation and technology undertaking” [1, 2], which is common in the biopharmaceutical field. The theoretical basis and research framework of relay innovation also focus on pharmaceutical enterprises. Obviously, the situation of the research object limits the applicability of the new innovation model of relay innovation. This study finds that, in addition to pharmaceutical enterprises, the phenomenon of relay innovation is still significant in high-tech fields such as high-end manufacturing, cloud computing, and artificial intelligence. For example, in October 2015, Google brain, Google's human intelligence team, developed tensor flow system to meet the needs of primary in-depth learning and application research; in 2018, Ant Finance obtained the authorization of the system program source code; the system was elastically reconstructed, so as to realize the stable online recommendation of Ant Finance's business scenario and greatly improve the processing capability of 10 billion feature vectors. It can be seen that the innovation achievements of heterogeneous innovation subjects are completely possible to realize innovation upgrading and cross domain application through the technological reengineering linkage of relay innovation. According to the research of Li et al. [3], the frequency of relay innovation was highly related to the complexity of knowledge system. Enterprises need to have diversified knowledge identification ability and intellectual property interest judgment ability, which refer to intellectual property capability. In order to explore the antecedent conditions and influencing factors of relay innovation and explore the factors affecting the efficiency and effectiveness of relay innovation, this study takes knowledge matching as the mediating variable, sets relationship learning as the moderating variable, empirically reveals the internal function mechanism of the impact of intellectual property capability on relay innovation, and tries to examine the problem of relay innovation from the perspective of intellectual property rights, so as to seek the new paths to improve the efficiency of technological innovation of unicorn enterprises. The theoretical research results and empirical analysis research results in this study will provide theoretical reference, theoretical basis, theoretical framework, and methodology system for the function mechanism of intellectual property capability on relay innovation with the help of the corresponding mediating variable and moderating variable, which will also provide empirical analysis basis and logical system, practical enlightenment, and decision-making reference for enhancing and promoting relay innovation with the help of intellectual property capability.

## 2. Relevant Literature Review

### 2.1. Relay Innovation

**Figure 1:**
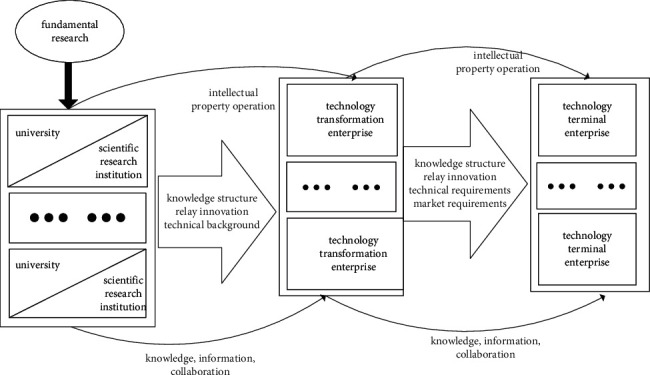
General enterprise relay innovation model.

The concept of relay innovation evolved from the “scientific business” model. In the research process of innovation heterogeneity, Pavitt first proposed that some innovation activities in high-tech enterprises were “based on science,” which required enterprises to have a large number of organized R&D activities closely related to science [4]. At the same time, Mowery believed that, in some industrial innovation with rapid technological progress, the cooperative innovation network formed by enterprises, universities, and government laboratories was the important place for the realization of innovation project [5]. According to the research of Link, the innovation subjects of biopharmaceutical enterprises successively included universities, public scientific research institutions, and enterprises. These organizations establish close ties and jointly participate in innovation in the innovation process [6]. Gibbons et al. further explained the interactions between scientific research and enterprise innovation in the process of relay innovation and explained the phenomenon that universities assumed enterprise functions and enterprises assumed academic functions described in the “three-helix” model of relay innovation [7]. According to Li et al. [8], relay innovation was an innovation mode characterized by sequential relay between existing and multiple subjects. In the standard relay innovation activity, different subjects with significantly heterogeneous capabilities and complementary advantages undertake the tasks of different links in the innovation chain, respectively, and complete the activities of conception, R&D, production, and commercial application in turn in the way of “relay;” finally, we realize the realization of innovation value. Platform technology transfer, overall sales, contract research, and market joint development all belong to the typical relay innovation model. According to the relevant research results [2], the general enterprise relay innovation model is shown in Figure 1.

### 2.2. Intellectual Property Capability

In the era of knowledge economy, intellectual property, as one of the important resources of enterprises, has taken the place of traditional tangible assets and occupied the important position in the development of enterprises [9]. Poltorak and Lerner proposed that the enterprise intellectual value chain was composed of intellectual capital, intellectual assets, and intellectual property rights, and intellectual property rights were at the high end of their value chains [10]. Intellectual property capability is a dynamic process of creating, applying, and managing intellectual property, and an important source of sustainable competitive advantage for enterprises [11]. According to Schilling, there was not a simple linear relationship between intellectual property rights and enterprise innovation performance [12]. In the process of organically combining their own resources with market demand, enterprises need to transform enterprise intellectual property rights into enterprise innovation performance by giving play to intellectual property creation, application, and synergy capabilities [12]. Tonisson et al. [13] and Alfred and Lothar [14] also believed that intellectual property capability provided new ideas and technologies for enterprise technological innovation and development by creating, processing, and realizing enterprise intellectual property value. Chi and Pan defined intellectual property capability as the comprehensive ability of enterprises to integrate physical resources and intellectual property, including effective mining, identification, transformation, application, and exchange of intellectual property [15].

### 2.3. Knowledge Matching

The matching theory originally derived from economics. The matching of economics refers to the structural equilibrium model that maximizes the interests of the paired parties in the collaborative environment based on the limited range of objects that can be selected by the supply and demand sides with a view to considering their respective goals and capabilities [16]. Tidd et al. introduced the matching theory in the field of economics into the field of management and proposed that the process of enterprise innovation was not only the process of managing the knowledge of enterprises, but also the process of continuously matching and integrating external knowledge and improving and expanding their own knowledge stock of enterprises [17]. Mindruta further explored the knowledge matching mechanism behind the formation of enterprise research cooperation from the perspective of knowledge resources according to the different knowledge attributes of knowledge supply and demand [18]. Yan and Bian start the research from knowledge complementarity, revealed how knowledge matching could meet the needs of both supply and demand of knowledge resources and transform knowledge resources into effective resources of enterprises, and divided knowledge matching into different levels of source knowledge, knowledge carrier, and application environment [19].

### 2.4. Relationship Learning

Hallen et al. proposed that relationship learning was an ability to establish relationships in the environment of cooperation and exchange with organizations [20]. Dyer and Singh believed that relationship learning was an important way for enterprises to create differentiation advantages and obtain excess profits in the process of communication with other organizations [21]. Selnes and Sallis put forward the concept of relationship learning from the perspective of information process and learning process. They believed that relationship learning belonged to the category of process learning; that is, in the process of joint learning, the partners are involved in learning and sharing information, deducing and integrating them into the specific shared relationship memory for storage [22]. Ling-Yee further extended the concept of relationship learning to the trade context. Their research showed that relationship learning meant that managers could share information and expand the specific relationship memory of the relationships with target customers and suppliers [23]. Chen et al. believed that collaborators related to enterprise relationship learning were distributed in all levels of units cooperating with enterprises, including customers, competitors, research institutions, and sales institutions [24]. Enterprises put emphasis on different organizational cooperation and learning and focus on different relational memory. Wang and Hsu embedded the concept of relationship learning into collective knowledge learning among partner enterprises, and partner organizations had an impact on collective learning through information sharing [25]. Based on the research of Selnes and Sallis, many scholars divided relationship learning into three dimensions of information sharing, common understanding, and relationship memory [25–28].

To sum up, the research on relay innovation is in its infancy. Scholars mostly focus on the description of the connotation and extension of relay innovation, emphasize the characteristics and functions of relay innovation, and initially form the theoretical framework system of relay innovation. However, few literature researches focus on the formation mechanism and influencing factors of relay innovation. At the same time, in the field of intellectual property research, some scholars have found that enterprise intellectual property capability can help enterprises effectively select external patents and technologies and increase the possibility of technology integration, absorption, and reengineering. Based on the above analysis, this study attempts to reveal the function mechanism of intellectual property capability on relay innovation. Starting with matching theory and learning theory, this study brings mediating variable knowledge matching and moderating variable relationship learning into the same conceptual model and theoretical analysis framework and tries to reveal the “black box” of the function mechanism of intellectual property capability on relay innovation and enrich and improve the research framework of relay innovation. Moreover, hybrid methods composed of fusion of rough set and binary firefly algorithm based on community weak connection mechanism (CWLBGSO), directed acyclic graph (DAG), and Bootstrap structural equation modeling (Bootstrap SEM) are used to carry out empirical analysis of the conceptual model and theoretical analysis framework of function mechanism of intellectual property capability on promoting the relay innovation performance of unicorn enterprises through the mediating role of knowledge matching and the moderating role of relationship learning.

## 3. Relevant Research Hypotheses

### 3.1. Intellectual Property Capability and Relay Innovation

Referring to the views of Cai et al. [29], this study divided intellectual property capability into three dimensions of intellectual property creation capability, intellectual property application capability, and intellectual property synergy capability. Intellectual property creation capability refers to the ability of enterprises to carry out intellectual property development and innovation through R&D investment and obtain intellectual labor achievements [30]. In previous studies, scholars generally believed that intellectual property creation capability played an important role in industrial innovation and development. For example, Robertson and Gatignon believed that intellectual property creation was the key to the commercialization development strategy of enterprises. The confirmation of intellectual capital, the creation of intellectual property, and industrial alliance network and cooperation are conducive to the diffusion and transmission of technological innovation [31]. Relay innovation is mainly composed of innovation discovery, innovation transformation, and innovation realization, while intellectual property creation is the core of relay innovation throughout [32]. In the innovation chain of relay innovation, universities, scientific research institutions, and other organizations with strong basic theoretical research ability are usually used as the front end of the innovation chain, while unicorn enterprises are responsible for technological innovation and market development as the middle and back end of the innovation chain [33]. As downstream organizations, unicorn enterprises with strong intellectual property creation capability often have strong opportunity discovery ability, effective technology identification ability, and information and knowledge search ability, which can help enterprises improve the utilization rate of intellectual property rights in the process of acquiring the knowledge of upstream organizations, carry out independent transformation and upgrading on the basis of the original intellectual property rights, and improve the performance of relay innovation while completing the objectives and tasks of relay innovation.

The intellectual property application capability is the ability to ensure the commercialization and marketization of intellectual property and bring benefits to enterprises through enterprises' own use, trusteeship, paid transfer, cross-licensing, and other means in the process of diffusion and transfer of intellectual property [26]. The application of intellectual property rights is the main way for the innovation subjects to make profits in the relay, and the transfer mode of intellectual property rights is the main means for the innovation subjects to realize the relay [32]. The improvement of intellectual property application capability helps downstream enterprises on the relay innovation chain choose appropriate intellectual property transfer methods, ensure that they can better connect with upstream organizations in the process of relay innovation, obtain more effective intellectual property with less resources, provide more choices and benefits for enterprises to participate in relay innovation, and promote the improvement of relay innovation performance of unicorn enterprises.

Intellectual property synergy capability refers to the ability of enterprises to share resources, information, and skills through intellectual property cooperation and exchange within and among industries in the context of open cooperation, so as to improve the comprehensive strength of enterprises [34]. Intellectual property synergy can increase access to external knowledge resources, make up for the lack of intellectual property rights in some fields, stimulate the creation of intellectual property rights, and then affect relay innovation [35]. In the process of relay innovation with other organizations, unicorn enterprises can keenly identify the advantages of other organizations and learn from each other's strengths to continuously interact with other enterprises in information, technology, and knowledge, in order to improve the performance of relay innovation. To sum up, this study puts forward the following hypothesis.

  Hypothesis 1: intellectual property capability has a positive impact on relay innovation.

### 3.2. Intellectual Property Capability and Knowledge Matching

Knowledge matching is the matching of knowledge in supply and demand. It depicts the knowledge coupling degree between the knowledge owner and the knowledge receiver, which is reflected in multiple levels such as source knowledge, application environment, and knowledge carrier [17]. Source knowledge matching requires that the transferred knowledge attributes need to meet the demand side of knowledge. If the knowledge to be transferred is tacit knowledge and has strong uniqueness and individuality, this kind of knowledge may not be effective knowledge for the demand side and may be excluded by the knowledge demand side [36, 37]. The openness and sharing of intellectual property synergy enable enterprises to fully understand the knowledge attributes of themselves and other enterprises in the process of cooperation and exchange, in order to provide more selectivity for knowledge matching. The application environment includes many market environment factors such as legal system, cultural atmosphere, and strategic decision-making [38]. The improvement of intellectual property synergy capability of unicorn enterprises makes them fully understand the application environment of other enterprises, so as to improve the success rate of knowledge matching [19].

Knowledge carrier matching is the matching between the existing form of knowledge owned by the knowledge owner and the recipient. Knowledge carriers can be divided into documents, equipment, codes, personal minds, and other forms. Similar knowledge carriers contribute to the rapid integration of heterogeneous knowledge. The improvement of the intellectual property application capability enables enterprises to introduce a variety of knowledge carriers in the process of intellectual property transformation, so as to accelerate the commercialization of intellectual property, enhance the diversity of knowledge carriers, and promote knowledge matching. In conclusion, the following hypothesis is put forward.

  Hypothesis 2: intellectual property capability has a positive impact on knowledge matching.

### 3.3. Knowledge Matching and Relay Innovation

Li et al. pointed out that relay innovation was easy to occur in industrial fields with complex knowledge system and high risk [3]. Relay innovation activities need to comprehensively use more complex cutting-edge technologies. Knowledge matching is the process for enterprises to improve the knowledge synergy and consistency between knowledge supply and demand through knowledge capture and knowledge absorption. Strong knowledge matching has an impact on the transformation and realization of relay innovation. The predesign and accurate matching of knowledge matching improve the availability and applicability of technical specific knowledge among organizations, help unicorn enterprises maintain familiarity and tacit understanding with relevant external knowledge sources, promote the rapid search of high-quality information and tacit knowledge [39], improve the screening and selection ability of enterprises for potential business achievements, and achieve the realization of relay innovation. In fact, knowledge matching can also be regarded as the verification and evaluation of the effectiveness of enterprise knowledge innovation infiltrated into the relay innovation link, which helps enterprises overcome the limitations of existing knowledge and practice, promote enterprises to distinguish, deconstruct, and reconstruct existing knowledge [40], enhance the adaptability and ease of use of enterprise knowledge, continuously activate and strengthen the internal innovation decision-making activities of enterprises, have the “refining” and “reengineering” of relay innovation achievements, shorten innovation time, and improve relay innovation quality, outcome, and efficiency. In conclusion, the following hypothesis is put forward.

  Hypothesis 3: knowledge matching has a positive impact on relay innovation.

### 3.4. The Mediating Variable of Knowledge Matching

Through the description of the relationships between intellectual property capability and knowledge matching, knowledge matching and relay innovation, it can be seen that intellectual property capability affects the relay innovation performance by affecting knowledge matching. Knowledge matching includes not only knowledge acquisition, but also the process of in-depth learning and exploration of the obtained knowledge, innovation in combination with their own situation, and bringing benefits to themselves [41]. The improvement of intellectual property capability of unicorn enterprises provides the suitable matching environment, knowledge support, and income guarantee for their knowledge matching. In the matching environment of symbiosis and mutual growth, unicorn enterprises use intellectual property creation to screen and identify effective knowledge resources and recreate them in combination with their own situations, so as to increase the process income of knowledge matching driven by the intellectual property application capability. According to the research of Muthusamy and White [42] and Gupta et al. [43], through various means such as knowledge transfer and absorption, knowledge matching could promote enterprises to obtain more effective resources externally and stimulate enterprises to effectively absorb and accumulate external knowledge resources, and then more innovative ways had emerged. Therefore, by improving the efficiency of knowledge matching, unicorn enterprises create the good innovation environment and many effective innovation methods for enterprise relay innovation, so as to improve the performance of relay innovation. In conclusion, the following hypothesis is put forward.

  Hypothesis 4: knowledge matching plays a mediating role between intellectual property capability and relay innovation.

### 3.5. The Moderating Effect of Relationship Learning

Jian et al. pointed out that relationship learning was an activity of sharing market information, common understanding, and knowledge dissemination among organizations [44]. This study believes that relationship learning is the organizational behavior for both parties of the cooperative enterprises to share, explain, and understand information based on business transactions and integrate it into the exclusive relationship memory. The essence of relationship learning is a learning process. The members participating in the process can share information, cooperate with each other, integrate knowledge and information and other resources, and finally form the exclusive cooperative memory in key areas to increase the interests of the members participating in relationship learning [45]. Based on the previous results of Chen and Cai [28] and Song et al. [45], this study divides relationship learning into three dimensions of information sharing, relationship memory, and common understanding. Yuan and Ning verified that the intensity of relationship learning helped organizations cultivate good communication relations and pay attention to the interactions between organizations in order to form the good organizational learning atmosphere [46]. Through relationship learning, organizations have strengthened information exchange, created the organizational support situation covering knowledge organization ability, organizational support ability, and service provision and guarantee ability [31], and improved the overall performance of enterprise knowledge absorption, intellectual property capability, and relay innovation.

Firstly, in the atmosphere of strong relationship learning, the ability of knowledge exchange and sharing between enterprises and partners is stronger. Enterprises participating in relationship learning can find ways to improve the quality, reliability, and speed of information transmission through information sharing [44]. At the same time, the specific relationship memory formed in the process of relationship learning is the information and experience accumulated by enterprises and partners through long-term interaction and cooperation, which affects the quality of partnership in the future. Therefore, strengthening the specific relationship memory can promote the improvement of intellectual property synergy and improve the effectiveness of knowledge acquisition and transfer in the process of knowledge matching, so that more knowledge can be effectively absorbed and utilized by enterprises.

Secondly, relationship learning is a process of strategic cooperation between enterprises and external organizations such as suppliers and scientific research institutions. The enhancement of relationship learning ability enables enterprises to timely obtain external information resources, understand the needs of the market, and quickly respond to market changes [31]. For enterprises with strong relationship learning, the specific relationship memory network is complex, and many partner enterprises participate in relationship learning. With the progress of relationship learning, partner organizations have the deeper understanding of each other [28], so as to improve the efficiency of information exchange among partner enterprises in the process of enterprise intellectual property coordination. As an important link in the process of relay innovation, information exchange directly affects the process of relay innovation.

Finally, relationship learning is a collective knowledge learning activity of learning and sharing experience between enterprises and partners [25]. Enterprises with strong relationship learning have stronger ability to transfer, acquire, and absorb knowledge resources, which are conducive to improving the matching degrees of knowledge between supply and demand. At the same time, strong relationship learning enterprises have strong common understanding ability, which is the adaptive adjustment ability between enterprises and cooperative enterprises [47], which can help enterprises improve the coupling degrees of knowledge in the process of knowledge matching, enrich enterprise knowledge resources, and promote the improvement of relay innovation performance. Based on the above analysis, the following hypotheses are put forward.

  Hypothesis 5: relationship learning has a positive moderating effect on intellectual property capability and knowledge matching; that is, in the strong relationship learning environment, intellectual property capability has a greater impact on knowledge matching, and vice versa.
  Hypothesis 6: relationship learning has a positive moderating effect on intellectual property capability and relay innovation; that is, in the strong relationship learning environment, intellectual property capability has a greater impact on relay innovation, and vice versa.
  Hypothesis 7: relationship learning has a positive moderating effect on knowledge matching and relay innovation; that is, in the strong relational learning environment, knowledge matching has a greater impact on relay innovation, and vice versa.

**Figure 2:**
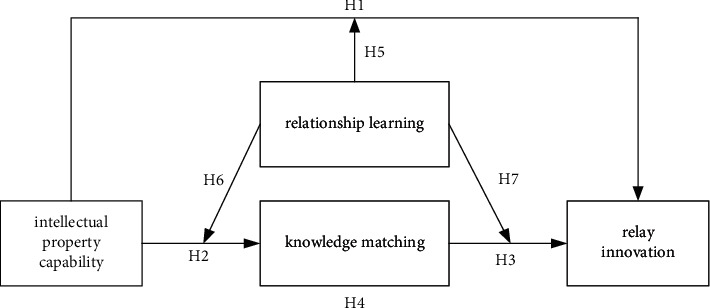
Conceptual model and theoretical analysis framework of function mechanism of intellectual property capability on relay innovation.

To sum up, this study constructs the conceptual model and theoretical analysis framework of function mechanism of intellectual property capability on relay innovation as shown in Figure 2, where the mediating variable is knowledge matching, and the moderating variable is relationship learning.

## 4. Research Design

### 4.1. Scale Development and Data Collection

In this study, the questionnaire is designed in the form of Likert five-level scales, in which the numbers from 1 to 5 indicate strongly disagree, disagree, general, agree, and strongly agree, respectively. In the process of data collection, firstly, the questionnaire items are analyzed by means of preinvestigation, and the items with poor identification ability, vague meaning, and scattered factor loading are deleted to form the formal questionnaire. According to the definition of unicorn enterprises by Lee [48], combined with the research of Chu and Song [49] and Cao [50], the following restrictions are made according to the actual needs of the survey: (1) the selected sample need to cover multiple high-tech fields such as Internet communication technology, biomedicine, and new materials and exclude enterprises with state-owned monopoly nature such as power enterprises and communication enterprises. (2) The respondents must be grass-roots managers, middle-level managers, and high-level managers of unicorn enterprises, with bachelor's degree or above and more than two years of working experience in the same industry. (3) Within the scope of controllable research time and cost, try to select sample enterprises in different regions to enhance the scientificity of the survey results. In this study, 270 questionnaires are distributed through field research, entrusted distribution, and e-mail, 246 questionnaires are recovered, 33 unqualified questionnaires are eliminated, and 213 valid questionnaires are finally obtained, with an effective rate of 78.9%. Descriptive statistics of the respondents showed that 48.8% of the respondents have bachelor's degree, 42.3% have master's degree, and 8.9% have doctorate; at the position level of enterprises, 51.6%, 39.0%, and 9.4% of the respondents are grass-roots managers, middle-level managers, and high-level managers; in terms of working years in the enterprises, 36.6% have worked for 2–5 years, 56.3% have worked for 5–10 years, and 7.1% have worked for more than 10 years.

The scale design mainly draws lessons from the mature items that have been used in the related literature and references, and some variables are adjusted and modified appropriately according to the actual needs of the survey. Based on the research context and intellectual property capability evolution of Cai et al. [29], intellectual property capability is divided into three dimensions of intellectual property creation capability, intellectual property application capability, and intellectual property synergy capability, with a total of 15 items. Based on the research of Li et al. [51], relay innovation is divided into three dimensions of innovation discovery, innovation transformation, and innovation realization, with a total of 8 items. Based on the research of Hu [52], knowledge matching is measured from three dimensions of source knowledge matching, knowledge carrier matching, and application environment matching, with a total of 8 items. Relationship learning is adopted by the research results of Chen and Cai [28], which is measured from three dimensions of information sharing, relationship memory, and common understanding, with a total of 15 items.

### 4.2. Reliability and Validity Analysis

This study adopts SPSS 21.0 software to carry out exploratory factor analysis of all variables, overall Cronbach's *α* coefficient is 0.835, indicating that the scale has good reliability, and other subvariables of Cronbach's *α* coefficients are above 0.6. In addition, Harman single factor test is used to test the common method deviation of the data. The test results indicate that KMO value is 0.924, and Bartlett spherical test is significant. Further, exploratory factor analysis of all variables shows that there is no common method deviation, which means that all variables show good discriminant validity. In conclusion, the items and data used in this study have good internal consistency. The results of Table 1 indicate that the scales have good reliability and validity, the values of Cronbach's *α* are all higher than 0.6, and the values of factor loading are all higher than 0.5.

Based on the hypotheses proposed in this study, the correlation coefficients among variables are tested. As shown in Table 2, there are significant positive correlations among intellectual property capability, relay innovation, relationship learning, and knowledge matching, and the significance levels (*p* value) corresponding to the correlation coefficients are less than 0.01.

The diagonals in Table 2 are the AVE square roots of latent variables. It can be seen that each AVE square root in this study is greater than the correlation coefficient with other latent variables. In order to further test the discriminant validity of variables, this study uses AMOS and MUPLUS software to conduct confirmatory factor analysis on the four variables in the conceptual model and theoretical analysis framework (intellectual property capability, relay innovation, knowledge matching, and relationship learning). As shown in Table 3, when the model of four factors is adopted, the data fitting effect is the best, indicating that the variables and scales have good validity and reliability.

## 5. Empirical Analysis Results

### 5.1. Empirical Analysis Methods

This study mainly involves three empirical analysis methods, which are divided into preliminary pretest and in-process test. Preliminary pretest is the premise and basis of in-process test. The three empirical analysis methods have logical relationships among each other, which constitute the logical context chain of mutual progression and show the progressive relationships among each other. The empirical analysis methods are involved in the fusion of rough set and binary firefly algorithm based on community weak connection mechanism (CWLBGSO), directed acyclic graph (DAG), and Bootstrap structural equation modeling (Bootstrap SEM). CWLBGSO and DAG are used in the preliminary pretest, and Bootstrap SEM is used in the in-process test.

Firstly, this study uses CWLBGSO to preliminarily identify the function mechanism of intellectual property capability on relay innovation and the interaction and mutual causality among variables in the conceptual model and theoretical analysis framework of function mechanism of intellectual property capability on relay innovation in advance, preliminarily verify the effectiveness and rationality of the conceptual model and theoretical analysis framework of function mechanism of intellectual property capability on relay innovation, carry out the necessity test and necessary condition test of conceptual model and theoretical analysis framework of function mechanism of intellectual property capability on relay innovation, and conduct the necessity test and necessary condition test of direct and indirect key core influencing factors and antecedent conditions (intellectual property capability, knowledge matching, and relationship learning) of relay innovation in the conceptual model and theoretical analysis framework of function mechanism of intellectual property capability on relay innovation (Figure 1).

Secondly, on the basis of adopting the CWLBGSO to pretest the direct and indirect influencing factors and antecedent conditions of relay innovation in the conceptual model and theoretical analysis framework of function mechanism of intellectual property capability on relay innovation, DAG is further adopted to preliminarily test the causal relationships among the independent variable (intellectual property capability), mediating variable (knowledge matching), moderating variable (relationship learning), and dependent variable (relay innovation) in the conceptual model and theoretical analysis framework of function mechanism of intellectual property capability on relay innovation. DAG is also used to preliminarily determine the directional causality, directional relationships, action relationships, and action directions among variables in the conceptual model and theoretical analysis framework of function mechanism of intellectual property capability on relay innovation. But DAG method fails to effectively test the main effect theoretical hypothesis, mediating effect theoretical hypothesis, and moderating effect theoretical hypothesis involved in the conceptual model and theoretical analysis framework of function mechanism.

Thirdly, on the basis of adopting DAG to preliminarily determine the sufficient conditions and causality, we adopt Bootstrap SEM to empirically test the main effect, mediating effect, and moderating effect of the conceptual model and theoretical analysis framework of function mechanism of intellectual property capability on relay innovation in order to empirically reveal the function mechanism of intellectual property capability on relay innovation through the mediating function of mediating variable (knowledge matching) and the moderating function of moderating variable (relationship learning).

### 5.2. Empirical Analysis Processes and Results

#### 5.2.1. Preliminary Pretest Based on CWLBGSO

Fusion of rough set and binary firefly algorithm based on community weak connection mechanism (CWLBGSO) effectively integrates the advantages of both of them [53–58].The main principles and modeling steps of fusion of rough set and binary firefly algorithm based on community weak connection mechanism (CWLBGSO) are as follows [53, 59–66].

Firstly, we present basic theoretical knowledge:

*(1) (Attribute) Reduction Optimization Objective*. Domain  *S*=*U*, *A*, *V*, *f* is defined as an expression system, and domain *U* is a collection of objects. Attributes is defined in *A*=*C* ∪ *D*, *C* represents condition, and *D*  represents decision. The attribute value is defined with *V*=*U*_*reR*_*V*_*r*_, and the value range is defined according to *V*_*r*_, and the value range of *r* ∈ *R* is defined. The information function is defined by *f* : *U* × *A*⟶*V* , and *U* mainly represents the attribute value of *x*. *S*=(*U*, *A*) is the set, *B*  ⊆  *A*, *B* ≠ ∅, and ∩*B* represent the intersection, and *B* in *IND*(*B*) represents the indistinguishable relationship. *S*=*U*, *A*,  *V*, *F* is information system. In *X* ⊆ *U* and *B* ⊆ *A*, the indistinguishable relationship of *IND*(*B*) in *x* is [*X*]_*B*_. Regulation *U* includes two approximation sets. Approximation set *B*^−^(*X*) is *B*^−^(*X*)={*x* *|* (*x* ∈ *U*∧[*x*]_*B*_∩*X* ≠ ∅)} and approximation set *B*_−_(*X*) is *B*_−_(*X*)={*x* *|* (*x* ∈ *U*∧[*x*]_*B*_ ⊆ *X*)}; further, *BN*_*B*_(*X*)=*B*^−^(*X*) − *B*_−_(*X*) represents the boundary of *X* on *B*. *k*=(*U*,  *R*) is the required approximate space, *P*, *Q* ∈ *R*, and knowledge *Q* and *P* on *k*(0 ≤ *k* ≤ 1) are expressed as *P*⇒*Q*.

(1) $$\begin{array}{c} k=r_{p}\left(Q\right)=\frac{\text{card}\left(pos_{P}\left(Q\right)\right)}{\text{card}\left(U\right)}. \end{array}$$

In the above formula, the set cardinality is card, and *pos*_*P*_(*Q*) is a *P* positive field of *Q*.

The objective function is obtained as

(2) $$\begin{array}{c} F\left(\wedge \right)=\frac{L-l}{L}+k. \end{array}$$

In the above formula, *L* represents the number of factors, *l* represents the number of attributes in the subset, and *k*(0 ≤ *k* ≤ 1) represents the dependency between attributes.

*(2) Attribute Dependency Solving Algorithm (ASDM)*. The ASDM algorithm can effectively solve the attribute dependency. The approximate space is explained by *k*=(*U*, *R*), the knowledge is explained by *P*, *Q* ∈ *R*, and *k*(0 ≤ *k* ≤ 1) is the dependence of *P* by *Q*. Solve the attribute card() with the same nature, and *k*=*r*_*P*_(*Q*) = {number of elements-{same condition attribute} ∩ {same nature attribute}}/{number of objects}.

Secondly, we present binary firefly optimization algorithm.

(1) Basic firefly algorithm:
The brightness of fireflies and the degree of spontaneous aggregation between individuals are important factors for the spontaneous aggregation of fireflies. Judging whether the obtained values are at the better level mainly depends on observing their location, and their location is determined by brightness. In addition, their brightness also further affects the degree of attraction. Individuals with darker fluorescence tend to those with brighter fluorescence nearby. If we judge the size of attraction, we need to observe the brightness of their fluorescence. If the movement of fireflies is irregular, it can be found that the brightness of the surrounding individuals is at similar level. The stronger the fluorescence, the smaller the degrees of aggregation among individuals, and the distances among individuals gradually decrease, which further absorb the fluorescence in the medium, resulting in the decrease of brightness.
At time *t*, fluorescein of firefly *θ* is
  (3) $$\begin{array}{c} l_{\theta }\left(t\right)=\left(1-\rho \right)l_{\theta }\left(t-1\right)+\mu J\left(x_{\theta }\left(t\right)\right). \end{array}$$
At time *t*, *θ* represents the distribution probability of *δ* moving in the specified dynamic set domain:
  (4) $$\begin{array}{c} P_{\theta \delta }\left(t\right)=\frac{l_{\delta }\left(t\right)-l_{\theta }\left(t\right)}{\sum_{keN_{\theta }\left(t\right)}l_{k}\left(t\right)-l_{\theta }\left(t\right)}. \end{array}$$
At time (*t* + 1), the position of firefly *θ* after random movement is
  (5) $$\begin{array}{c} x_{\theta }\left(t+1\right)=x_{\theta }\left(t\right)+s^{*}\left[\frac{x_{\delta }\left(t\right)-x_{\theta }\left(t\right)}{\left\|x_{\delta }\left(t\right)-x_{\theta }\left(t\right)\right\|}\right]. \end{array}$$
At time *t* , the radius of firefly *θ* dynamic set domain is updated:
  (6) $$\begin{array}{c} r_{d}^{\theta }\left(t+1\right)=\min\left\{r_{s},\max\left\{0,r_{d}^{\theta }\left(t\right)+\beta \left(n_{t}-\left|N_{\theta }\left(t\right)\right|\right)\right\}\right\}, \end{array}$$
where the volatilization concentration and change rate are expressed by *ρ* and *μ*, the number is expressed by *n*_*t*_, *r*_*s*_ is the moving radius, and *β* represents the update rate.
(2) One-dimensional binary cellular automata model:
There are only two states for the corresponding variables of the binary discrete optimization problem, namely, the corresponding variable (expressed by 1) or the noncorresponding variable (represented by 0). Ten-bit binary codes are listed according to the two states to obtain the *L*-dimensional 0/1 sequence. *L* represents the length of the cell matrix, *θ* represents the individual in the firefly, and the cell value is *Q* ∈ {0,1}.
Adopt the formula *s*(*x*_*θr*_)=1/1+exp(−*x*_*θr*_), constrain the firefly position among 0/1, and *x*_*θr*_(*t*+1) meets the following constraints:
  (7) $$\begin{array}{c} x_{\theta r}\left(t+1\right)=\left\{\begin{array}{c} 0,sign\left(x_{\theta r}\left(t\right)\right)\leq 0.5,\\ 1,sign\left(x_{\theta r}\left(t\right)\right)>0.5. \end{array}\right. \end{array}$$
According to formulas (3)–(7), the cells of fireflies are randomly selected to obtain the 0/1 cell array. The continuously changing firefly community is discretized according to the Tent mapping mechanism.

Thirdly, we present binary firefly algorithm based on weak connection mechanism optimization

**Figure 3:**
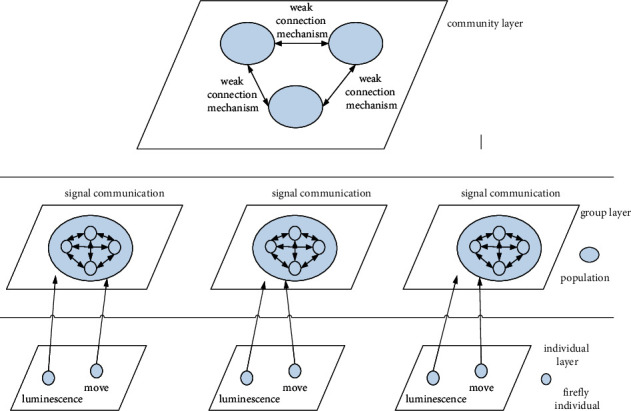
Optimization hierarchy model based on biological population behavior.

(1) Weak connection mechanism: the results of independently searching for fireflies are divided into multiple subpopulations. Select the suboptimal individual in each subpopulation to interact, and calculate the optimal results combined with binary firefly algorithm.
(2) The division of search space and the initial solution of chaotic sequence: chaotic sequence based on the improved Tent is adopted. The whole search space (*Z*) is equally divided into *n* subintervals of the same sizes and paired with the corresponding subpopulation, and the following formula is obtained:
  (8) $$\begin{array}{c} \bar{x_{\theta r}}\left(t+1\right)=\left\{\begin{array}{c} 2\times \left(\bar{x_{\theta r}}\left(t\right)+0.1\times rand\left(0,1\right)\right),\;x_{\theta r}<0.5,\\ 2\times \left(1-\left(\bar{x_{\theta r}}\left(t\right)+0.1\times rand\left(0,1\right)\right)\right),\;0.5<x_{\theta r}\leq 1. \end{array}\right. \end{array}$$
The chaotic sequence position of Tent mapping is between (0,1), and the position of particles after mapping refers to
  (9) $$\begin{array}{c} x_{\theta r}\left(t\right)=\bar{x_{\theta r}}\left(t\right)\times \left(x\max_{\theta r}-x\min_{\theta r}\right)+x\min_{\theta r}. \end{array}$$
(3) Suboptimal interindividual interaction: the suboptimal individuals in different firefly populations are selected, and the selected firefly individuals interact to obtain new firefly populations with more quantity and better quality, so as to realize diversified firefly populations.
(4) Optimization hierarchy model based on biological community behavior: the optimization hierarchy model based on biological community behavior is shown in Figure 3.
(5) The main steps of CWLBGSO: the main steps of CWLBGSO are as follows:
  *Step 1*. Divide the whole search space into *n* subintervals with upper and lower boundary values, and pair each subinterval with the corresponding subpopulation.
  *Step 2*. Define and assign the initial value to the fluorescein of firefly itself, the decision domain determined by the radius, and the parameters of the individual itself, and assign the initial value to the location of each firefly by using the Tent chaotic sequence method.
  *Step 3*. According to one-dimensional binary cellular automata, firefly individuals are given 0 or 1: 1 represents the evaluation index of key factors, and 0 represents the evaluation index of nonkey factors. Further assign the main simulation parameters, XMIN={−5, −2.5, 0,2.5}, XMAX={−2.5, 0,2.5, 5}, fluorescein initial value is *l*_*i*_(0)=5.0, and fluorescein volatility is *ρ*=0.4, *μ*=0.6, *s*=0.35. Other relevant parameters need further continuous improvement, optimization, and adjustment according to the research problems.
  *Step 4*. After each iteration, calculate the new adaptation value generated by the corresponding 0/1 sequence of fireflies in each subpopulation.
  *Step 5*. Select the new suboptimal individual and calculate the new adaptation value of the suboptimal individual to the 0/1 sequence after mutual interaction.
  *Step 6*. By comparing with the populations after the interaction among the original subpopulation and the suboptimal individual, the optimal sequence fitness in the firefly population is found out.
  *Step 7*. Update the fluorescein and location of individual fireflies.
  *Step 8*. Update the decision domain radius of firefly individuals.
  *Step 9*. Repeat the cycle from step 3 to step 8 until the end condition is met.
  *Step 10*. Give out the outputs and results that meet the end conditions.

The purpose of CWLBGSO in this study is to preliminarily identify the function mechanism of intellectual property capability (intellectual property creation capability, intellectual property application capability, and intellectual property synergy capability) on relay innovation and the interaction and mutual causality among variables in the conceptual model and theoretical analysis framework of function mechanism of intellectual property capability on relay innovation in advance, preliminarily verify the effectiveness and rationality of the conceptual model and theoretical analysis framework of function mechanism of intellectual property capability on relay innovation, carry out the necessity test and necessary condition test of conceptual model and theoretical analysis framework of function mechanism of intellectual property capability on relay innovation, and conduct the necessity test and necessary condition test of direct and indirect key influencing factors and antecedent conditions (intellectual property capability, knowledge matching, and relationship learning) of relay innovation in the conceptual model and theoretical analysis framework (Figure 1) of function mechanism of intellectual property capability on relay innovation.

In order to further empirically verify the rationality of the key core influencing factors of relay innovation (intellectual property creation capability, intellectual property application capability, intellectual property synergy capability, knowledge matching, and relationship learning), the auxiliary influencing factors of relay innovation are added in the empirical analysis process of CWLBGSO, and the additional auxiliary influencing factors consist of ten elements. The dependent variable in the conceptual model and theoretical analysis framework of function mechanism is set as relay innovation. On the basis of the method principles and modeling steps and the optimization hierarchical model of biological population behavior, this study adopts the main principles and steps of CWLBGSO, integrates the questionnaire data of the previous variables and scales, sets the previous effective questionnaire samples (the number of effective questionnaire samples is 213) as the test data, follows the parameter setting of the above CWLBGSO algorithm, and uses MATLAB software to simulate the CWLBGSO algorithm, and the simulation identification results of the CWLBGSO algorithm show that the combined method selects five key core influencing factors of relay innovation, mainly including intellectual property creation capability, intellectual property application capability, intellectual property synergy capability, knowledge matching, and relationship learning. The reduction rate of influencing factors is 1–5/15 = 66.67%. The contribution rate and interpretation rate of five key core influencing factors are as follows: the optimal classification accuracy of the original dataset is 88.4625% (higher than the threshold value of 80%), the optimal classification accuracy of the subset of key core influencing factors is 89.2425% (higher than the threshold value of 80%), the difference of the optimal classification accuracy of key core influencing factors is 0.78%, the average classification accuracy of the original dataset is 81.4462% (higher than the threshold value of 80%), the average classification accuracy of the subset of key core influencing factors is 80.4427% (higher than the threshold value of 80%), and the average classification accuracy difference of the subset of key core influencing factors is 1.0035%. The CWLBGSO algorithm simulation results ensure that the conceptual model and theoretical analysis framework of function mechanism of intellectual property capability on relay innovation pass the necessity test and necessary condition test. The effectiveness and rationality of the conceptual model and theoretical analysis framework of function mechanism of intellectual property capability on relay innovation are preliminarily verified and demonstrated.

#### 5.2.2. Preliminary Pretest Based on DAG

Directed acyclic graph, also known as directed acyclic path graph, refers to a directed graph with acyclic paths, that is, a graph in which all edges are directed edges and acyclic paths, which represents the asymmetric contemporaneous causality among sequences and variables [67–76].

**Figure 4:**
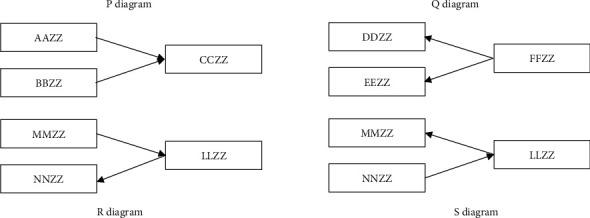
Multiple causalities among variables in DAG.

For ∀ AAZZ, BBZZ, CCZZ in V, if AAZZ-BBZZ, then AAZZ and BBZZ are called neighbors. If AAZZ⟶BBZZ, then AAZZ is called the parent node of BBZZ, then AAZZ is the direct cause of BBZZ, BBZZ is a child node of AAZZ, and then BBZZ is the direct result of AAZZ. If AAZZ to BBZZ has a directed path (e.g., AAZZ ⟶ CCZZ ⟶ BBZZ), then AAZZ is the grandfather node of BBZZ; that is, AAZZ is the cause of BBZZ caused by CCZZ, BBZZ is the descendant node of AAZZ, and then BBZZ is the result of AAZZ through CCZZ. In the DAG model, if there is ⟶ CCZZ ⟵ on path *π*, point CCZZ is called the collision point on path *π*; otherwise, it is called the noncollision point. If the following two points are met, (1) each noncollision point on path *π* is not in set *S* and (2) each collision point on path *π* is in set *S*, then path *π* is said to be connected by set *S*_*d*−_; otherwise, it is said to be blocked by *d*−. If all paths between AAZZ and BBZZ are blocked by set *S*_*d*−_, AAZZ and BBZZ are isolated by set *S*_*d*−_. For example, for a set of ∀ variables *W* = {AAZZ, BBZZ, CCZZ, DDZZ, EEZZ, FFZZ, LLZZ, MMZZ, NNZZ}, the multiple causality among variables in DAG is shown in Figure 4. According to the above definition, in Figure 4 P diagram, variables AAZZ and BBZZ are not related to each other; that is, the conditional correlation coefficient is 0. Therefore, the empty set *φ* is a separate set between variables AAZZ and BBZZ. CCZZ is the result of AAZZ and BBZZ. It is the collision point between AAZZ and BBZZ. Therefore, set *W* = {CCZZ} does not isolate all paths d between AAZZ and BBZZ; that is, set {CCZZ} is not an isolated set between AAZZ and BBZZ. As for *Q* diagram in Figure 4, FFZZ is the common cause of DDZZ and EEZZ and the noncollision point on the path of DDZZ and EEZZ. Therefore, set {FFZZ} isolates all paths d between DDZZ and EEZZ; that is, set {FFZZ} is the isolation set between DDZZ and EEZZ. As for *R* diagram and *S* diagram in Figure 4, causality is a causal chain; that is, MMZZ is the cause of LLZZ, and LLZZ is the cause of NNZZ. Or NNZZ is the cause of LLZZ and LLZZ is the cause of MMZZ. The unconditional correlation coefficient between MMZZ and NNZZ is not zero. LLZZ is the noncollision point on the path between MMZZ and NNZZ, and set {LLZZ} isolates all paths d between MMZZ and NNZZ; that is, set {LLZZ} is the isolation set between MMZZ and NNZZ [67, 77–84].

DAG mainly analyzes the directivity of multiple repeated and miscellaneous causalities among research variables based on d-isolation set (d-separation). According to the above definition of d-isolation set (d-separation), for any variable set *W* = {AAZZ, BBZZ, CCZZ}, AAZZ and CCZZ, CCZZ and BBZZ are neighbors to each other (the correlation coefficient is significantly different from 0): (1) if AAZZ and BBZZ are not adjacent (the correlation coefficient is not significant) and CCZZ is not in the separation set of AAZZ and BBZZ, DAG is determined as in *P* diagram in Figure 4. (2) If the direction between AAZZ and CCZZ is known to be AAZZ ⟶ CCZZ, and CCZZ is in the separation set of AAZZ and BBZZ, then DAG is determined as in *Q* diagram in Figure 4. (3) If the DAG determined according to the above method is not unique, it shall be judged according to the economic and management situation, and the macrosituation, mesosituation, and microsituation shall be taken as the selection basis. On the above significance test, the *Z* statistic of Fisher is applied to test whether the conditional correlation coefficient is significantly not equal to zero [67, 85–90]:

(10) $$\begin{array}{c} Z\left(p\left(i,j\left|k\right|\right),n\right)=\left(\frac{1}{2}\sqrt{n-\left|k\right|-3}\right)\times \ln\left(\frac{1+\rho \left(i,j\left|k\right|\right)}{1-\rho \left(i,j\left|k\right|\right)}\right), \end{array}$$

where *n* is the number of samples, *ρ*(*i*, *j*|*k*|) is the overall conditional correlation coefficient of sequences *i* and *j* with respect to sequence *k*, and |*k*| is the number of variables. If sequences *i*, *j*, and *k* obey the normal distribution and *γ*(*i*, *j*|*k*|) is the sample conditional correlation coefficient of sequences *i* and *j* with respect to sequence *k*, then *Z*(*ρ*(*i*, *j*|*k*|), *n*) − *Z*(*γ*(*i*, *j*|*k*|), *n*) obeys the standard normal distribution.

In this study, d-isolation set (d-separation) is combined with PC algorithm to determine the contemporaneous causality among variables. Firstly, the complete graph among variables is determined; that is, there is an undirected edge between any two variables; secondly, the unconditional correlation coefficients among variables are calculated, and the edges with insignificant correlation coefficients are eliminated; thirdly, the first-order partial correlation coefficient shall be tested in the remaining undirected edges, and so on. If there are *N* variables, the *N* – 2 partial correlation coefficient should be calculated at most; finally, the directivity of undirected edges in the graph is determined by using the method of d-separated set [67–73].

**Figure 5:**
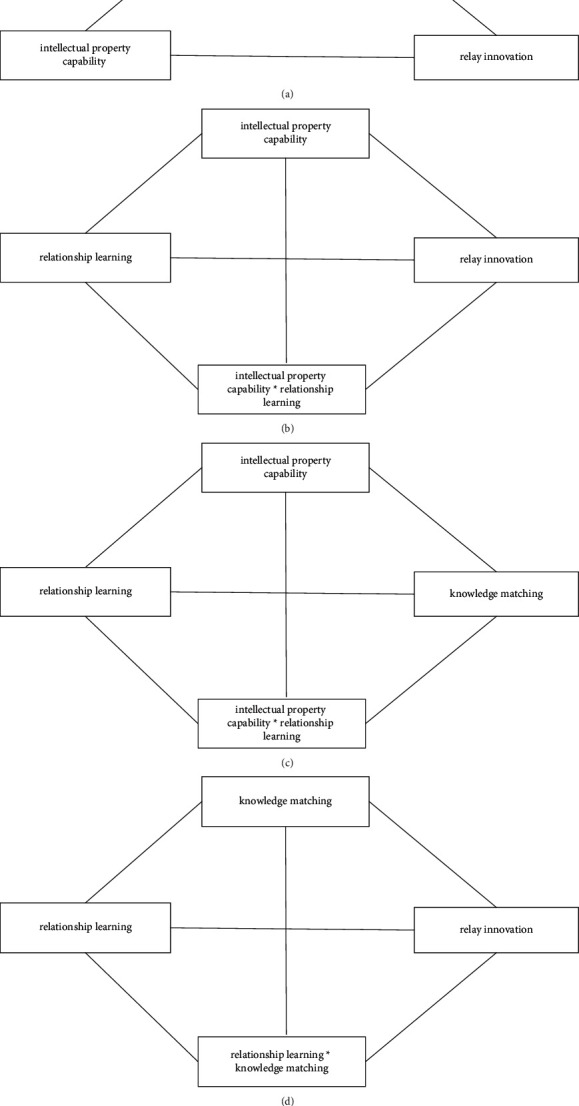
Empirical analysis of undirected complete graph. (a) Causal relationships between intellectual property capability and relay innovation based on mediating variable of knowledge matching in undirected complete graph. (b) Causal relationships between intellectual property capability and relay innovation based on moderating variable of relationship learning in undirected complete graph. (c) Causal relationships between intellectual property capability and knowledge matching based on moderating variable of relationship learning in undirected complete graph. (d) Causal relationships between knowledge matching and relay innovation based on moderating variable of relationship learning in undirected complete graph.

**Figure 6:**
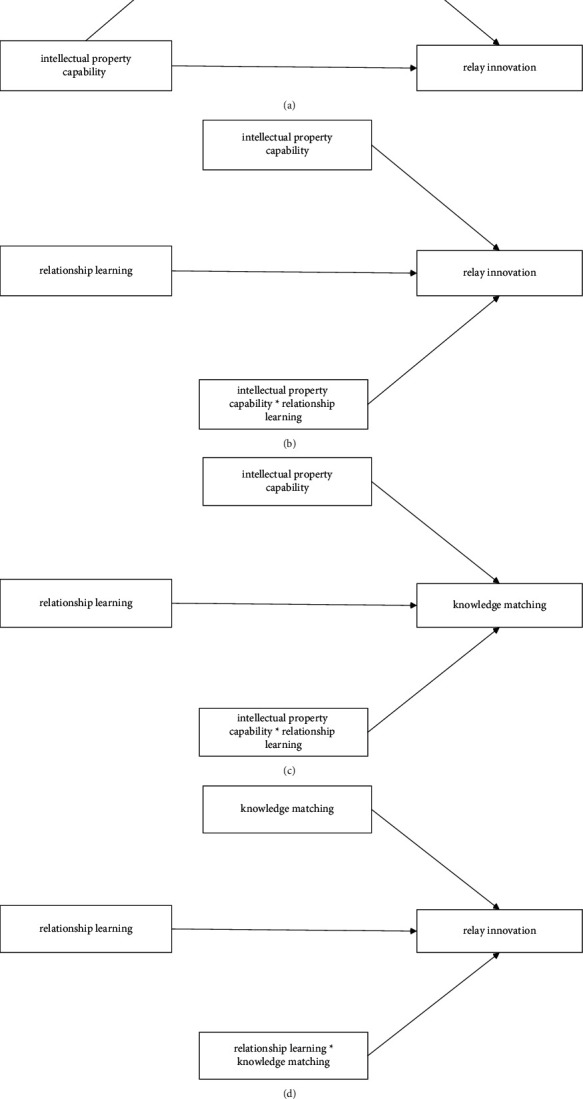
Empirical analysis of DAG. (a) Causal relationships between intellectual property capability and relay innovation based on mediating variable of knowledge matching in DAG. (b) Causal relationships between intellectual property capability and relay innovation based on moderating variable of relationship learning in DAG. (c) Causal relationships between intellectual property capability and knowledge matching based on moderating variable of relationship learning in DAG. (d) Causal relationships between knowledge matching and relay innovation based on moderating variable of relationship learning in DAG.

This study adopts DAG method with the help of Tetrad IV software and executes PC algorithm in order to verify the multiple repeated and miscellaneous causal relationships among independent variable, mediating variable, moderating variable, and dependent variable in the conceptual model and theoretical analysis framework of function mechanism of intellectual property capability on relay innovation. The relevant DAG empirical analysis results are shown in Figures 5 and 6 (undirected complete graph and directed acyclic graph), and the directivity results among independent variable, mediating variable, moderating variable, and dependent variable in Figures 5 and 6 further pretest and verify the directivity, guidance, effectiveness, and necessity of relevant theoretical research hypotheses in the conceptual model of function mechanism of intellectual property capability on relay innovation in advance and further preliminarily demonstrate the multiple repeated and miscellaneous causalities among variables in the conceptual model and theoretical analysis framework of function mechanism of intellectual property capability on relay innovation in Figure 1.

#### 5.2.3. Empirical Analysis of Function Mechanism Based on Bootstrap SEM

*(1) Mediating Effect Analysis*. The AMOS 23.0 and MUPLUS 7.0 software are used to carry out empirical analysis of Bootstrap structural equation modeling (Bootstrap SEM) to verify and test the theoretical hypotheses. The total amount of Bootstrap sample is 2000. The structural equation modeling method is used to analyze the data, and the results are shown in Table 4. It can be seen that intellectual property capability has a significant positive influence on relay innovation (model 1: *β* = 0.857, *p* < 0.001). Among them, intellectual property creation capability, intellectual property application capability, and intellectual property synergy capability all have significant positive impacts on relay innovation (*β*1 = 0.692, *p* < 0.001; *β*2 = 0.596, *p* < 0.001; *β*3 = 0.656, *p* < 0.001), and hypothesis 1 is verified.

The mediating effect is tested according to the mediating effect test procedure proposed by Wen et al. [91]. Model 2 and model 3 integrate mediating variable into the structural equation modeling to test the relationships between independent variable and dependent variable, respectively, the relationships between independent variable and mediating variable, and the relationships between independent variable and dependent variable under the action of mediating variable. The results in Table 4 show that intellectual property capability has a significant positive impact on knowledge matching (*β* = 0.612, *p* < 0.01), and hypothesis 2 is verified; knowledge matching has a significant positive impact on relay innovation (*β* = 0.277, *p* < 0.01), and hypothesis 3 is verified. The results obtained in Table 4 show that, after the introduction of mediating variable knowledge matching, although the influence coefficient value of intellectual property capability on relay innovation *β* decreases, the influence is still significant (*β* = 0.687, *p* < 0.01), indicating that knowledge matching plays a mediating variable, and hypothesis 4 is tested.

**Figure 7:**
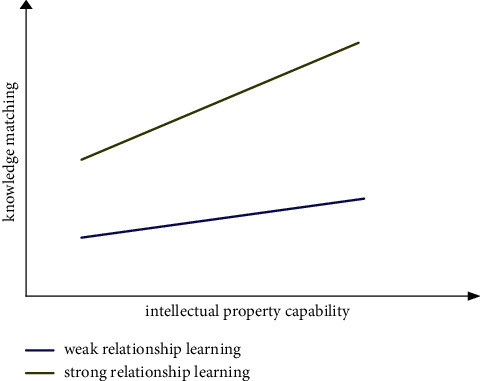
The moderating effect of relationship learning on intellectual property capability and knowledge matching.

**Figure 8:**
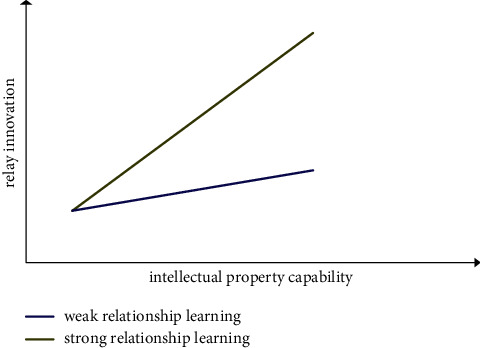
The moderating effect of relationship learning on intellectual property capability and relay innovation.

**Figure 9:**
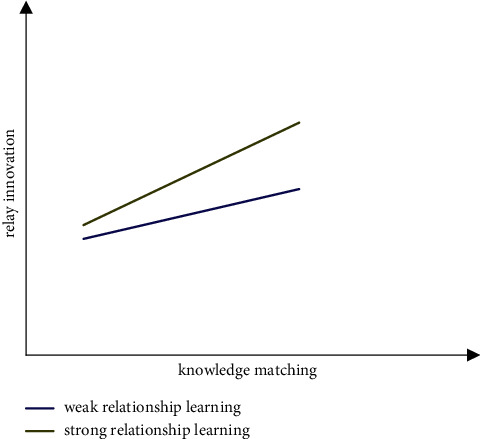
The moderating effect of relationship learning on knowledge matching and relay innovation.

*(2) Moderating Effect Analysis*. According to the moderating effect test procedures proposed by Wen et al. [92], this study determines the interactive items in model 5, model 7, and model 9 before verifying the moderating effect. Interactive item 1 is intellectual property capability *∗* relationship learning; interaction item 2 is knowledge matching *∗* relationship learning. Then, the variables used are centralized; that is, the variables subtract their mathematical expectations, so as to eliminate the multicollinearity problem among the interactive items and the main variables in the conceptual model and theoretical analysis framework. The variance expansion factor (VIF value) is used to test the multicollinearity among variables. The VIF values are less than value 4, indicating that there is no obvious multicollinearity among variables. At the same time, DW statistics are used to test the autocorrelation of residuals to eliminate its impact on the prediction function of the model. The values of DW statistics are all around 2, indicating that there is no autocorrelation problem in the model. Referring to sequential test methods for moderating effects of Muller et al. [93], we test the moderating effects. According to the structural equation modeling test of intellectual property capability, knowledge matching, moderating variable, and interactive item (intellectual property capability *∗* relationship learning) in model 4 and model 5 in Table 4, it is shown that relationship learning, as the moderating variable, plays a positive moderating role between intellectual property capability and knowledge matching (*β* = 0.234, *p* < 0.05), and as shown in Figure 7, the stronger the enterprise relationship learning, the stronger the influence of intellectual property capability on knowledge matching. Hypothesis 5 is verified. Model 6 and Model 7 conduct structural equation modeling tests on intellectual property capability, relay innovation, moderating variable, and interactive item (intellectual property capability *∗* relationship learning) and show that relationship learning, as the moderating variable, plays a positive moderating role between intellectual property capability and relay innovation (*β* = 0.316, *p* < 0.05), and as shown in Figure 8, the stronger the enterprise relationship learning, the stronger the impact of intellectual property capability on relay innovation, and hypothesis 6 is verified. Models 8 and 9 carry out structural equation modeling tests on knowledge matching, relay innovation, moderating variable, and interactive item (knowledge matching *∗* relational learning), showing that relationship learning, as the moderating variable, plays a positive moderating role between knowledge matching and relay innovation (*β* = 0.218, *p* < 0.05), and as shown in Figure 9, the stronger the enterprise relationship learning, the stronger the influence of knowledge matching on relay innovation. Hypothesis 7 is verified. The necessary goodness of fit index in Table 4 reaches the specified standard and exceeds the specified threshold, the ratio of Chi-square to degree of freedom is greater than value 1 and less than value 3, RMSEA is lower than 0.08, and CFI and TLI are all higher than 0.9. In Table 4, *N* = 213, ^*∗*^indicates *p* < 0.05, ^*∗∗*^indicates *p* < 0.01, and ^*∗∗∗*^indicates *p* < 0.001.

## 6. Research Conclusions

Relay innovation is an important innovation mode to comply with the explosive growth of unicorn enterprises. Through relay innovation, enterprises can identify effective innovation opportunities and reduce innovation risks. Intellectual property capability is an important means to improve patent and technology transformation efficiency and reduce technological innovation cycle. This study focuses on the new innovation mode with the main manifestations of technology undertaking and sequential relay of unicorn enterprises and puts emphasis on the origin of relay innovation. Taking knowledge matching and relationship learning as the starting points, this study constructs the conceptual model and theoretical analysis framework of function mechanism of intellectual property capability on relay innovation through the mediating effect of knowledge matching and the moderating effect of relationship learning, makes out the empirical analysis study, and draws the following main conclusions and enlightenment. Hybrid methods composed of fusion of rough set and binary firefly algorithm based on community weak connection mechanism (CWLBGSO), directed acyclic graph (DAG), and Bootstrap structural equation modeling (Bootstrap SEM) can orderly carry out empirical analysis of the conceptual model of the function mechanism, and the hybrid methods can successfully and effectively reveal and expound the function mechanism of intellectual property capability on relay innovation through the mediating effect of knowledge matching and the moderating effect of relationship learning. The empirical analysis results show that intellectual property capability has a positive role in relay innovation of unicorn enterprises significantly, knowledge matching plays the positive mediating role between the relationships of intellectual property capability and relay innovation significantly, relationship learning plays the moderating role of the relationships between intellectual property capability and relay innovation, relationship learning plays the moderating role of the relationships between intellectual property capability and knowledge matching, and relationship learning plays the moderating role of the relationships between knowledge matching and relay innovation.

Previous studies had shown that the level of intellectual property capability was an important factor in determining the innovation mode adopted by enterprises, and the improvement of intellectual property capability brought about a variety of possibilities for enterprise innovation. For example, Namvar et al. [94] and Barney [95] believed that, from the perspective of resource view theory, the competitive advantage of enterprises originated from internal heterogeneous resources, and the intellectual property capability just conformed to the above characteristics. However, relevant studies generally followed the traditional intellectual property management process to define the content of intellectual property capability; that is, they believed that intellectual property capability included the creation, application, protection, and management of intellectual property, and they put emphasis on the importance of intellectual property system [96]. As the unicorn enterprise with capital agglomeration and technology agglomeration, the interaction and reprocessing of information, knowledge, and technology are obviously particularly important for relay innovation. According to the needs of the research topic, this study regards intellectual property synergy as an important part of intellectual property capability and finds that intellectual property synergy can dilute the complexity of relay innovation knowledge system and alleviate the contradiction of intellectual property distribution in all linkages of relay innovation and improve the frequency and success rate of relay innovation. The research conclusion suggests that unicorn enterprises need to balance the relationships between intellectual property synergy and intellectual property protection. Reasonable intellectual property synergy can create the appropriate intellectual property application scenario and make various tacit knowledge elements of the “transferee” of relay innovation explicit. Reasonable intellectual property synergy can also stimulate the innovation inspiration of unicorn enterprises and reduce the enterprise learning obstacles caused by heterogeneous knowledge.

In the process of information exchange and technology transfer among relay innovation subjects, knowledge matching ability is particularly important. This study puts forward the logical framework of “intellectual property capability-knowledge matching-relay innovation,” takes knowledge matching as an important path of the internal influence mechanism of intellectual property capability and relay innovation, and verifies the mediating effect of knowledge matching. According to the research results of Wilkesmann et al. [97] and Rhodes et al. [98], knowledge matching reflects the unity of the usefulness and ease of use of knowledge, and the effectiveness of knowledge matching depends on the ability of the “transferee” to construct, deconstruct, and reconstruct the source knowledge. The research conclusion points out that knowledge matching itself belongs to the dynamic matching process from “learning application feedback”; from the perspective of professional division of labor, it is necessary for unicorn enterprises to introduce professional individuals or organizations to assume the role of “knowledge gatekeeper,” improve the efficiency and outcome of knowledge acquisition, analysis, screening, and processing and the matching degrees of knowledge, and finally enhance the innovation effectiveness of unicorn enterprises.

Common relay innovation cooperation methods include technology licensing, data authorization, commissioned research, etc. Unicorn enterprises with strong relationship learning have stronger ability to understand, absorb, and transform knowledge or technology, which are conducive to enterprise knowledge exchange, so as to make it easier for enterprises to obtain knowledge matching and relay innovation achievements. This study takes relationship learning as the moderating variable to adjust, moderate, and strengthen the relationships among intellectual property capability, knowledge matching, and relay innovation, respectively. This conclusion is the further deepening achievements of the related research results of scholars Ruey and Rudolf [27], Chen and Cai [28], Jian et al. [44], and Powell et al. [99]; these scholars all take relationship learning as the independent variable to explore the mutual impact of relationship learning and enterprise innovation performance. This study believes that relationship learning, as one of the means of knowledge exchange and knowledge learning among enterprises, cannot directly affect the innovation performance of enterprises, which further affects the innovation performance of unicorn enterprises by adjusting, moderating, and strengthening the relationships among enterprise intellectual property capability, knowledge matching, and relay innovation, respectively. At the practical level, unicorn enterprises need to clarify the internal relationships among relationship learning, knowledge matching, and intellectual property capability. Relay innovation cooperation parties should improve the openness of knowledge learning and pursue higher-level relationship learning activities with the attitude of seeking common ground while reserving differences, so as to improve the effectiveness and outcome of intellectual property capability, knowledge matching, and relay innovation.

## Figures and Tables

**Table 1:** Reliability and validity analysis of the scales.

Variables	Dimensions	Observation variables	Factor loadings	Cronbach's *α*
Intellectual property capability	Intellectual property creation capability	V1	0.755	0.738
		V2	0.693	
		V3	0.686	
		V4	0.705	
	Intellectual property application capability	V5	0.738	0.782
		V6	0.752	
		V7	0.643	
		V8	0.624	
		V9	0.678	
	Intellectual property synergy capability	V10	0.626	0.762
		V11	0.722	
		V12	0.831	
		V13	0.709	
		V14	0.685	
		V15	0.702	

Relay innovation	Innovation discovery	V16	0.720	0.740
		V17	0.648	
	Innovation transformation	V18	0.800	
		V19	0.697	
		V20	0.655	
	Innovation realization	V21	0.717	
		V22	0.825	
		V23	0.743	

Knowledge matching	Source knowledge matching	V24	0.716	0.757
		V25	0.698	
		V26	0.724	
	Knowledge carrier matching	V27	0.784	
		V28	0.771	
		V29	0.699	
	Application environment matching	V30	0.787	
		V31	0.775	

Relationship learning	Information sharing	V32	0.689	0.723
		V33	0.699	
		V34	0.728	
		V35	0.807	
		V36	0.780	
		V37	0.672	
	Common understanding	V38	0.733	
		V39	0.711	
		V40	0.694	
		V41	0.743	
		V42	0.765	
	Relationship memory	V43	0.723	
		V44	0.695	
		V45	0.800	
		V46	0.739	

Note: relevant items are obtained from relevant literature and references.

**Table 2:** Descriptive statistics and correlations among variables.

Variables	Mean	SD	1	2	3	4	5	6
Intellectual property creation capability	3.482	0.307	**0.710**					
Intellectual property application capability	3.460	0.313	0.587^*∗∗*^	**0.688**				
Intellectual property synergy capability	3.428	0.295	0.693^*∗∗*^	0.582^*∗∗*^	**0.715**			
Relay innovation	3.456	0.378	0.624^*∗∗*^	0.518^*∗∗*^	0.580^*∗∗*^	**0.728**		
Knowledge matching	3.438	0.222	0.704^*∗∗*^	0.621^*∗∗*^	0.687^*∗∗*^	0.630^*∗∗*^	**0.743**	
Relationship learning	3.447	0.212	0.578^*∗∗*^	0.512^*∗∗*^	0.553^*∗∗*^	0.574^*∗∗*^	0.734^*∗∗*^	**0.733**

The diagonals in Table 2 are the AVE square roots of latent variables. The bold values in Table 2 mean the AVE square roots of latent variables.

**Table 3:** Confirmatory factor analysis (*N* = 213).

Model		X^2^/df (the ratio of chi-square to degree of freedom)	CFI	GFI	TLI	RMSEA
Four factors	IPC, RI, KM, RL	1.027	0.954	0.927	0.938	0.011
Three factors	IPC + RL, RI, KM	3.879	0.880	0.788	0.874	0.057
Three factors	IPC + KM, RL, RI	4.678	0.846	0.729	0.812	0.063
Three factors	IPC + RI, RL, KM	5.031	0.827	0.711	0.826	0.082
Two factors	IPC + KM, RL + RI	8.928	0.825	0.706	0.823	0.096
Two factors	IPC + KM + RL, RI	9.124	0.810	0.692	0.808	0.139
Single factor	IPC + RI + KM + RL	12.031	0.802	0.576	0.775	0.148

Note: IPC stands for intellectual property capability, RI stands for relay innovation, KM stands for knowledge matching, and RL stands for relationship learning.

**Table 4:** Test results of mediating effect and moderating effect.

Variables	Mediating effect			Moderating effect					
	Relay innovation	Relay innovation	Knowledge matching	Knowledge matching	Knowledge matching	Relay innovation	Relay innovation	Relay innovation	Relay innovation
	Model 1	Model 2	Model 3	Model 4	Model 5	Model 6	Model 7	Model 8	Model 9
*Independent variables*									
Intellectual property capability	0.857^*∗∗∗*^	0.687^*∗∗*^	0.612^*∗∗*^	0.595^*∗∗*^	0.474^*∗*^	0.574^*∗∗*^	0.517^*∗*^		
Intellectual property creation capability	0.692^*∗∗∗*^								
Intellectual property application capability	0.596^*∗∗∗*^								
Intellectual property synergy capability	0.656^*∗∗∗*^								

*Mediating variable*									
Knowledge matching		0.277^*∗∗*^						0.645^*∗∗*^	0.707^*∗*^
Moderating variable									
Relationship learning				0.156^*∗*^	0.717^*∗*^	0.368^*∗*^	0.310^*∗*^	0.245^*∗*^	0.309^*∗*^

*Moderating effect*									
Interactive item (intellectual property capability *∗* relationship learning)					0.234^*∗*^		0.316^*∗*^		
Interactive item (knowledge matching *∗* relationship learning)									0.218^*∗*^
The ratio of chi-square to degree of freedom	2.812	2.736	2.589	2.601	2.667	2.698	2.678	2.536	2.482
RMSEA	0.075	0.072	0.059	0.061	0.063	0.068	0.067	0.057	0.054
CFI/TLI	0.910/0.908	0.914/0.913	0.928/0.924	0.925/0.923	0.924/0.922	0.917/0.915	0.922/0.920	0.933/0.929	0.937/0.932

## Data Availability

The datasets used and/or analyzed during the current study are available from the corresponding author upon reasonable request.
